# Inhibition as a Binary Switch for Excitatory Plasticity in Pyramidal Neurons

**DOI:** 10.1371/journal.pcbi.1004768

**Published:** 2016-03-22

**Authors:** Katharina A. Wilmes, Henning Sprekeler, Susanne Schreiber

**Affiliations:** 1 Department of Biology, Institute for Theoretical Biology, Humboldt-Universität zu Berlin, Berlin, Germany; 2 Bernstein Center for Computational Neuroscience, Berlin, Germany; 3 Technische Universität Berlin, Berlin, Germany; Hamburg University, GERMANY

## Abstract

Synaptic plasticity is thought to induce memory traces in the brain that are the foundation of learning. To ensure the stability of these traces in the presence of further learning, however, a regulation of plasticity appears beneficial. Here, we take up the recent suggestion that dendritic inhibition can switch plasticity of excitatory synapses on and off by gating backpropagating action potentials (bAPs) and calcium spikes, i.e., by gating the coincidence signals required for Hebbian forms of plasticity. We analyze temporal and spatial constraints of such a gating and investigate whether it is possible to suppress bAPs without a simultaneous annihilation of the forward-directed information flow via excitatory postsynaptic potentials (EPSPs). In a computational analysis of conductance-based multi-compartmental models, we demonstrate that a robust control of bAPs and calcium spikes is possible in an all-or-none manner, enabling a binary switch of coincidence signals and plasticity. The position of inhibitory synapses on the dendritic tree determines the spatial extent of the effect and allows a pathway-specific regulation of plasticity. With appropriate timing, EPSPs can still trigger somatic action potentials, although backpropagating signals are abolished. An annihilation of bAPs requires precisely timed inhibition, while the timing constraints are less stringent for distal calcium spikes. We further show that a wide-spread motif of local circuits—feedforward inhibition—is well suited to provide the temporal precision needed for the control of bAPs. Altogether, our model provides experimentally testable predictions and demonstrates that the inhibitory switch of plasticity can be a robust and attractive mechanism, hence assigning an additional function to the inhibitory elements of neuronal microcircuits beyond modulation of excitability.

## Author Summary

We must constantly learn in order to meet the demands of a dynamically changing environment. The basis of learning is believed to be synaptic plasticity, i.e. the potential of neuronal connections to change. Depending on context, however, it may be either useful to learn and modify connections or, alternatively, to keep an established network structure stable to maintain what has been already learned (also referred to as the plasticity-stability dilemma). The ability to switch synaptic plasticity on and off in a flexible way hence constitutes an attractive feature of neuronal processing. Here, we analyze a cellular mechanism based on the inhibition-mediated gating of coincidence signals required for Hebbian forms of excitatory synaptic plasticity. While experimental evidence in support of individual steps involved in this mechanism is accumulating, it is as of now unclear whether this mechanism can indeed operate robustly under physiologically realistic parameters of pyramidal cells, in particular, without impairing information flow in these cells altogether. Computational modeling allows us to demonstrate that this is indeed possible if inhibition is well timed (on the order of 1 ms). Moreover, we show that a specific design of the local circuit can ensure the necessary timing.

## Introduction

To successfully interact with our environment, we need to adjust to new or changing conditions. It is widely accepted that this ability involves alterations of synaptic connections in the brain, so-called synaptic plasticity [1]. While synaptic plasticity fulfills key requirements for the incorporation of new knowledge and memories into neural circuits, it also introduces the risk of changing connections that are essential for previously stored information, a problem termed plasticity-stability dilemma [2, 3]. Hence, a mechanism to selectively switch plasticity on or off would be useful.

In this context, inhibition has been proposed as a means to regulate plasticity [4–6]. GABAergic interneurons that target the dendrites of pyramidal cells may control backpropagation of action potentials to excitatory synapses and hence the coincidence signal required for Hebbian forms of synaptic plasticity. Alternatively, such inhibition could affect the NMDA-receptor-mediated component of excitatory postsynaptic potentials (EPSPs) [5, 7]. Indeed, recent experimental work has shown that inhibitory dendritic synapses can weaken the backpropagating action potential (bAP) in the dendrite of pyramidal cells so that calcium signals required for the plasticity of excitatory synapses are reduced [8]. These findings provide strong support for a crucial role of inhibition as a regulator of plasticity, in particular as direct stimulation of inhibitory neurons leads to a cancellation of bAPs in pyramidal cells. For an effective switch in plasticity, however, it needs to be ensured that while the dendritic inhibition cancels the bAP, the forward-directed EPSP (that is meant to cause the bAP in the first place) still reaches the soma and orthodromic information flow is preserved. Whether a separation of the effect of inhibition on EPSP and bAP is possible on physiological timescales is currently unclear and needs to be explored in view of the well-known efficiency of ‘on-path’ inhibition in impairing passive EPSPs in the dendrite [9]. We here address this question as well as identify the temporal and spatial constraints that are required to reliably modulate plasticity of excitatory synapses in pyramidal cells.

To this end, mathematical modeling suggests itself, because it allows to systematically vary both the strength and dendritic location of inhibition and to monitor bAPs and calcium spikes in the whole dendrite with high temporal and spatial resolution. We hence adapted a multi-compartmental model of pyramidal cells to reproduce a number of key electrophysiological characteristics. These included a realistic decrease of the amplitude of bAPs along the dendritic tree [10], characteristics of bAP-activated calcium spike (BAC) firing [11], and the generation of distal calcium spikes at a critical frequency of somatic stimulation [12]. Using this model, we identify conditions under which a modulation of bAPs and calcium spikes is possible and does not impair the forward-directed information flow to the soma.

We demonstrate that shunting inhibition gates bAPs and calcium spikes in an all-or-none manner, laying the foundation for a binary switch of plasticity of excitatory synapses. Further, we identify the timing constraints for an efficient inhibitory gating of bAPs and calcium spikes. Implementing an additive spike-timing-dependent plasticity (STDP) rule [13], we find that plasticity of excitatory synapses is indeed switched as a consequence of the effects of inhibition on the coincidence signal. Depending on the site of inhibition, this effect can be more global or constrained to smaller dendritic compartments and hence also distinct pathways converging onto pyramidal neurons. We observe that timing constraints for the gating of bAPs are relatively strict in that inhibition has to arrive with a precision of ∼1 ms. Local gating of distal calcium spikes, however, can be achieved within a wider time window of several milliseconds. Additionally, calcium spikes are more sensitive to inhibition in that smaller conductances suffice to abolish them. Importantly, with appropriate timing of inhibition, forward-directed EPSPs can still drive somatic firing while Hebbian coincidence signals are canceled. Finally, we suggest that a common circuit motif of feedforward inhibition can ensure the appropriate timing required for the plasticity switch by inhibition. Our model study provides testable experimental predictions and strengthens the view that the functional role of interneurons includes a pathway-specific regulation of plasticity, in addition to the widely studied regulation of excitability and information flow in local networks [14].

## Results

Pyramidal cells form one of the most important classes of projection neurons in the mammalian brain. Their apical and basal dendrites often receive synaptic inputs that originate from different pathways [15]. Accordingly, these cells are an appealing target for pathway-specific computations. Here, we focus on cortical layer 5 and hippocampal CA1 pyramidal neurons. Besides extending over multiple layers, these neuron types share physiological properties [15] such as attenuating bAPs [16] and the generation of calcium spikes in the apical tuft (see e.g. [17, 18]. Hebbian plasticity of excitatory synapses in these cells is assumed to require a coincidence signal that provides information about the occurrence of somatic spikes to dendritic synapses. The hypothesis central to our study is that well-timed and localized inhibition can selectively cancel bAPs or calcium spikes and hence impair synaptic plasticity at excitatory synapses further down the dendritic tree because of the missing coincidence signal.

**Fig 1:**
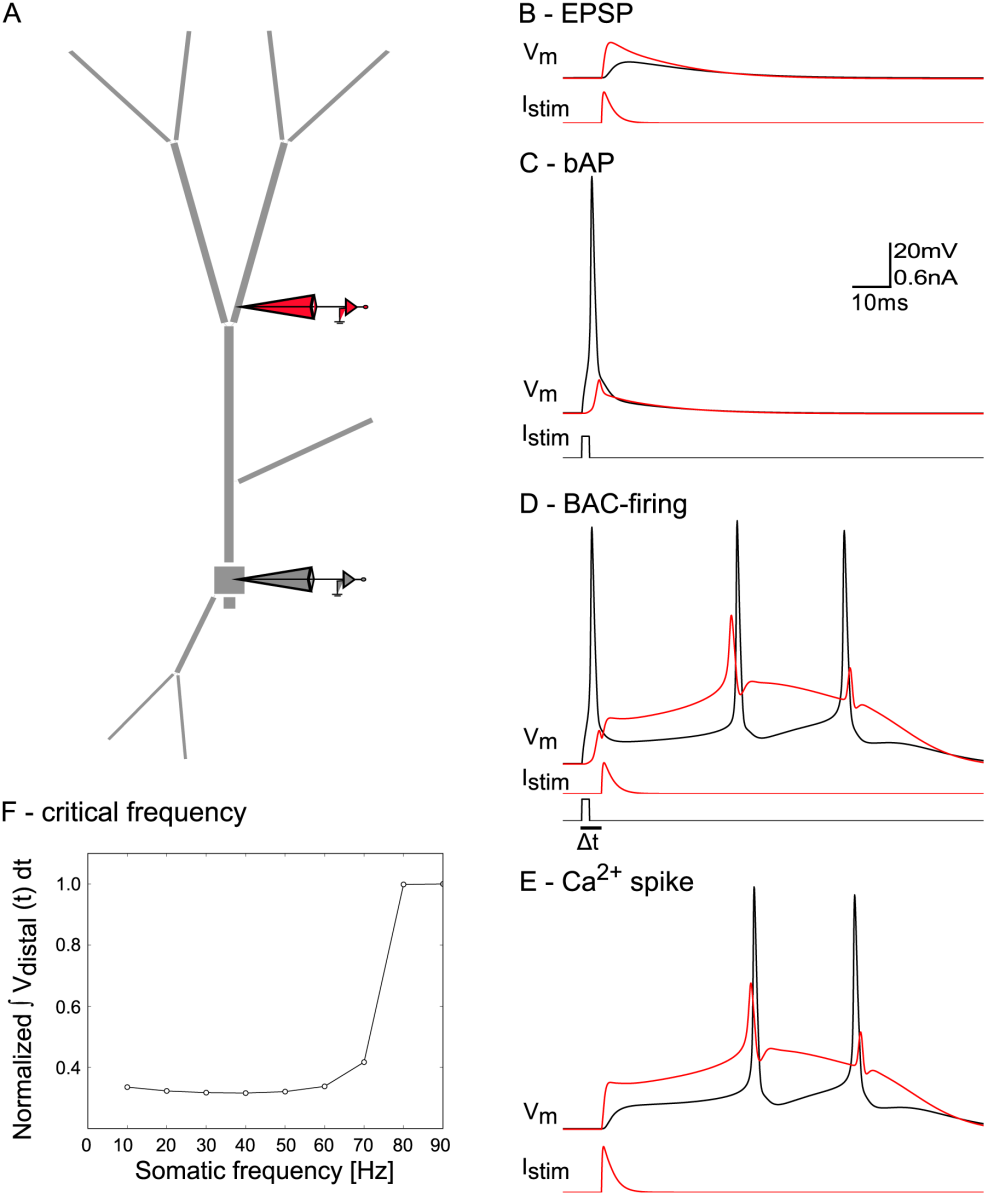
Response properties of simplified pyramidal neuron model. The model reproduces qualitative features of pyramidal neuron dendrites found in experiments by [11] and [12]. The color of the voltage traces match the electrodes in the diagram. Scale bar applies to panels B-E. A: Model neuron morphology with somatic (black) and dendritic (red) recording and stimulation sites. B: EPSP: a distal EPSC (I_stim_) resulted in an EPSP in the dendrite with little effect on somatic voltage. C: bAP: threshold somatic current injection (0.3 nA, 2 ms) led to a bAP. D: BAC firing: somatic current injection followed by dendritic stimulation in an interval (Δt) of 5 ms, resulted in a calcium spike, and a burst of two somatic APs. E: Calcium spike: stronger dendritic stimulation alone could elicit a calcium spike. F: Above a critical frequency of somatic spiking, a calcium spike was triggered. The y-axis depicts the cumulative membrane potential across the fixed simulation length of 0.6 sec for different frequencies of somatic stimulation. All values are normalized by the value at 90 Hz stimulation frequency (where a stereotypical calcium spike was elicited). The distal membrane potential changed abruptly once the frequency was high enough to trigger the calcium-dependent dendritic nonlinearity (i.e. the calcium spike).

We investigated the feasibility of such inhibition-mediated control of plasticity in a multi-compartmental model neuron with a simplified morphology capturing basic features of the dendritic compartments (including apical, oblique, and basal dendrites), as well as the soma and the axon (see Fig 1A). Electrophysiological and biophysical parameters were chosen in physiologically realistic ranges and carefully adjusted to exhibit well-know properties of pyramidal cells. The model neuron captured a realistic decrease of the amplitude of bAPs along the dendritic tree [10], characteristics of BAC firing [11], as well as the generation of distal calcium spikes at a critical frequency of somatic stimulation [12]. Specifically, the neuron generated attenuating bAPs (Fig 1C) upon somatic current injection, and calcium spikes in the presence of coincident synaptic input in the distal part of the apical trunk (Fig 1D), while distal input alone did not suffice to trigger a calcium spike (Fig 1B). Calcium spikes in turn gave rise to a burst of two somatic action potentials. Such bursts have been observed in pyramidal neurons in hippocampal CA1 [17, 19], and cortical layer 5 (BAC firing, [11]). Hence, dendritic input modulated the gain of the somatic f-I curve. When stimulated to fire with increasing frequency, the model neuron produced a dendritic nonlinearity at a critical frequency of 80 Hz (Fig 1F).

Inhibition was assumed to be shunting with rise and decay time constants of 0.5 ms and 5 ms, respectively. This kind of inhibition is commonly observed in pyramidal neurons receiving inhibitory GABA_A_ synapses [20] and it acts locally [21, 22]. Finally, we also demonstrate that our main results generalize to an anatomically reconstructed and physiologically detailed L5 neuron model [23].

### All-or-none modulation of backpropagating action potentials by inhibition

**Fig 2:**
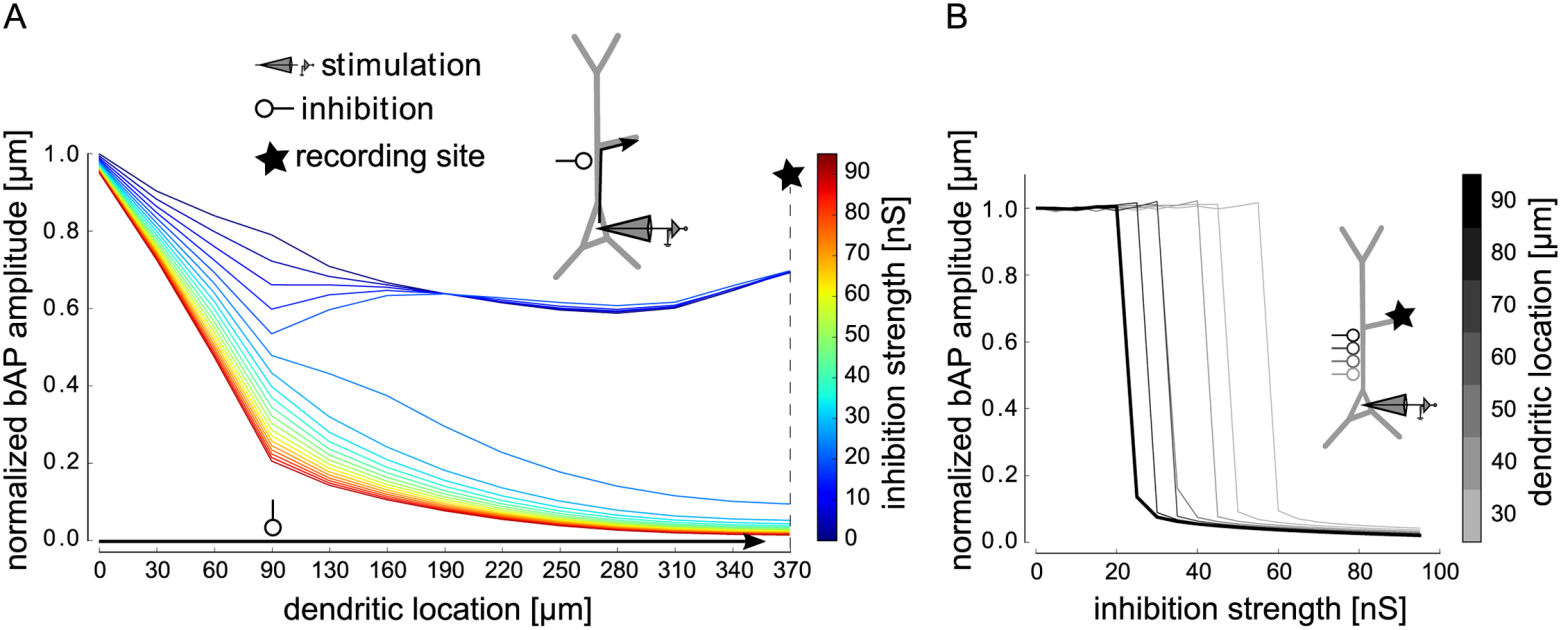
Analysis of the effect of inhibition on the backpropagating action potential (bAP) in dependence on inhibitory conductance and dendritic location. The bAP was triggered by threshold somatic current injection (as in Fig 1C). A: Amplitude of the bAP on its path from the soma, along the proximal apical dendrite into the distal oblique dendrite (arrow), normalized to its amplitude at the soma, for different values of inhibition strength (lines in different colors). Inhibition with a double exponential time course (τ_rise_ = 0.5 ms, τ_decay_ = 5 ms) was placed on the proximal apical dendrite (90 μm from the soma). Inhibition onset was 2 ms after stimulation onset. The somatic spike peaked around 2.5 ms after stimulation onset. B: Amplitude of the inhibited bAP relative to the non-inhibited bAP as a function of inhibition strength, measured in the distal part of the oblique dendrite (indicated by star in illustration and in A) for different locations of inhibition on the apical trunk (different shades indicate distance to soma in μm, thick line for 90 μm). Inhibition had an all-or-none effect on the bAP.

First, we sought to understand the effect of local dendritic inhibition on bAPs. To this end, we placed a shunting inhibitory input on the proximal apical dendrite, systematically varied its strength, and monitored the amplitude of a somatically-induced bAP along the apical dendrite. For weak inhibition, the bAP amplitude was reduced around the dendritic location of the inhibitory input, but recovered to its full amplitude as the bAP propagated down the dendrite. In contrast, strong inhibition barred the bAP from invading the dendrite (Fig 2A). The transition from a fully intact bAP to a complete bAP failure occurred abruptly at a critical inhibitory conductance (Fig 2B). This all-or-none behavior was observed for inhibition at different locations along the shaft of the apical dendrite, albeit with different critical amounts of inhibition (Fig 2B). From the perspective of a synapse further up in the dendrite, the bAP was either fully intact or completely canceled. Fig 2 shows the case for a synapse on the oblique dendrite, which is taken as a representative for one excitatory pathway subject to bAP-dependent STDP throughout the paper. The same effect could, however, also be observed for propagation into the apical tuft. The amount of inhibition required to cancel a bAP appeared to be in a realistic range. For example, when inhibition was placed at 90 μm from the soma (Fig 2A and 2B), the critical inhibitory conductance was slightly above 25 nS, corresponding to about 25 co-activated inhibitory synapses of 1 nS [24]. Such inhibition could be provided by about 2–3 co-activated interneurons, given that interneurons form up to a dozen contacts onto a single pyramidal cell [25]. We checked that the core observations of the effects of inhibition onto bAPs were not altered when shunting synaptic inputs were distributed along the dendrite (S1 Fig).

A moderate temporal jitter (on the order of 1 ms) in the activation of inhibitory synapses also did not abolish the all-or-none behavior, but when a critical amount of inhibition was reached too early, the neuron was prevented from spiking. Overall, however, timing of inhibition needed to be relatively precise, as we will outline in the following section, where we first present data on the compartment-specificity of the effects.

### Compartment-specific inhibition

Due to its relatively local effect, shunting inhibition can control dendritic signals in a manner that is distinct between compartments, such as basal and apical dendrites. We hence investigated the effect of inhibition in different compartments (i.e. the basal, oblique and distal apical dendrites, as well as the soma/axon), on the bAPs and calcium spikes, in the same and other compartments.

#### Modulation of bAPs

**Fig 3:**
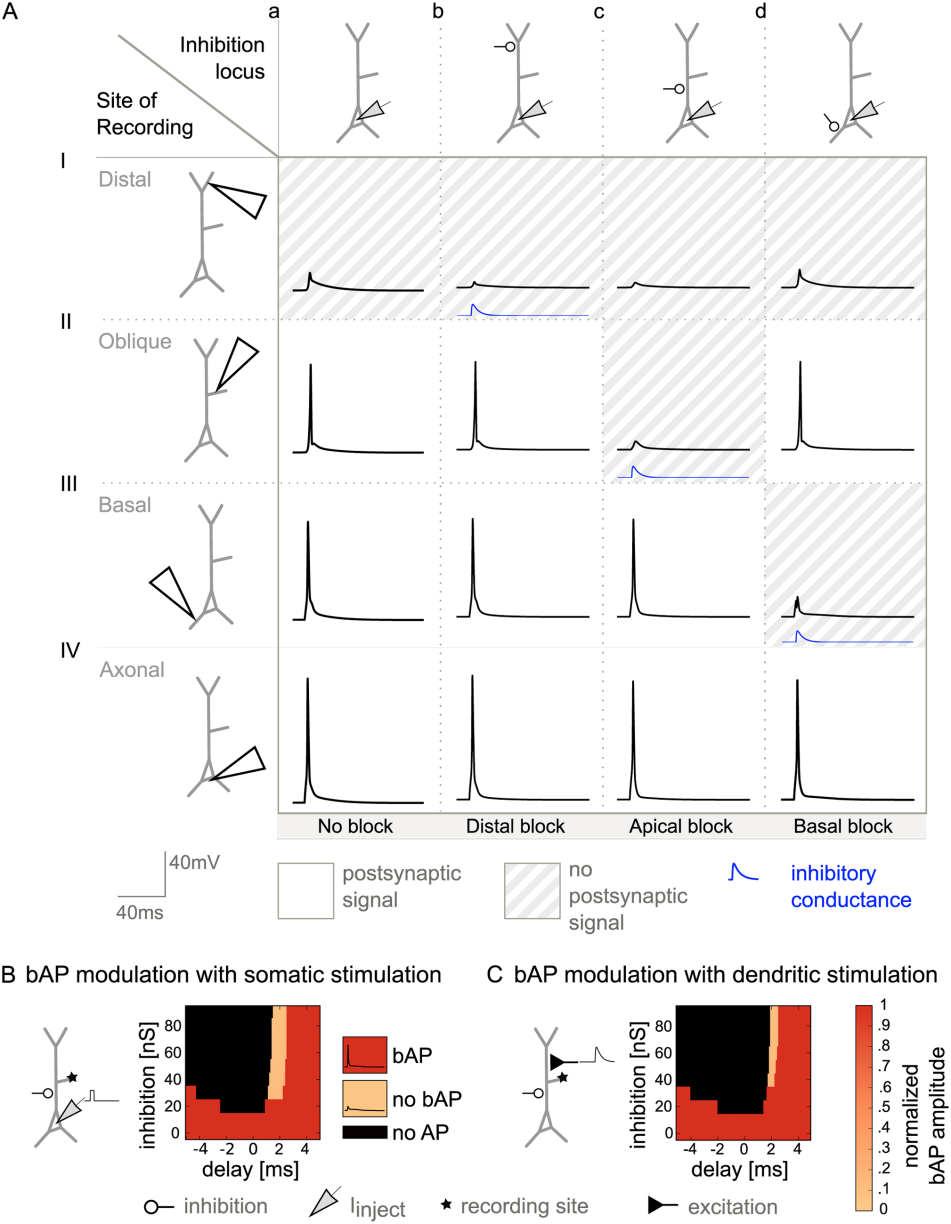
Compartment-specific inhibition of bAPs in the absence of calcium spikes. A: Effect of inhibition locus on bAPs, elicited by somatic current injection. The recording site varies along the rows; the site of inhibition varies along the columns. Inhibitory conductance is indicated by the blue traces (inhibition onset was 1.5 ms after stimulation onset.). a: No inhibition. b: Distal inhibition (460 μm, 50 nS) affected the distal bAP, but did not have a pronounced effect in the absence of a calcium spike (I), it however left signaling in the remaining dendritic tree intact (II and III). c: Proximal inhibition (90 μm, 50 nS) affected signaling in the whole apical dendrite by eliminating the bAP (II), but did not affect the bAP in the basal dendrite (III). d: The bAP in the basal dendrite, and thus basal plasticity, was suppressed by basal inhibition (100 μm, 50 nS) (III), while apical signaling was unchanged (I and II). B: Effect of inhibition onset timing on bAP and calcium spike modulation. Inhibition was shunting with GABA_A_ time constants (τ_rise_ = 0.5 ms, τ_decay_ = 5 ms). Strength of inhibition varies along each y-axis, onset of inhibition relative to the onset of the somatic step current varies along each x-axis. A: Proximal inhibition on the apical dendrite and its effect on bAPs. The corresponding somatic APs were elicited by somatic step current injection (as in Fig 1C) and peaked around 2.5 ms after stimulation onset. Color-coded is the amplitude of the bAP measured in the oblique dendrite (star indicates recording site). Unless somatic spiking was inhibited (black), either a full-blown bAP (red) or no bAP (light orange) could be observed. C: The neuron was driven by excitatory synapses distributed along the apical trunk to represent inputs from oblique dendrites (see Methods). Modulation of the bAP was possible in a narrow time window.

First, we consider the general case of bAP modulation in the absence of BAC firing. A systematic illustration of the effects of shunting inhibition on bAPs in different compartments is presented in Fig 3A. We placed the inhibitory input in different locations and recorded the membrane potential dynamics throughout the dendrite to detect bAPs. Each panel shows the local membrane potential as a function of time in the absence (a) or presence (b-d) of shunting inhibition. The location of the shunting inhibition varies across the columns of the figure, the site of recording of the voltage trace varies across rows. Compartments where the local bAP was canceled by inhibition (i.e. the remaining voltage amplitude was small) are shaded in gray.

The first column in Fig 3A lays out the voltage traces in the control situation without inhibition, showing an AP in the axon initial segment and related bAPs in the other compartments. Distal inhibition further decreased the already severely attenuated bAP in the distal dendrite, but did not change signaling in the apical oblique and basal dendrites (Fig 3A and 3b). With inhibition onto the proximal dendrite, the bAP could be barred from the apical dendrite, without affecting the bAP in the basal dendrite (Fig 3A–3c). Finally, basal inhibition canceled the bAP in the basal dendrite (Fig 3A–3d).

#### Timing requirements for bAP modulation

To assess whether timing requirements for the modulation of bAPs are realistic, we varied both the strength and the timing of proximal inhibitory inputs and monitored their effect. As discussed above, bAPs were canceled by inhibition in an all-or-none manner. We hence distinguished three cases: (1) inhibition had no effect on the bAP, (2) inhibition canceled the bAP in the dendrite without affecting the somatic spike (the scenario of interest for the modulation of plasticity), and (3) somatic firing was abolished altogether and hence also no bAP was observed. The sensitivity of the bAP to proximal inhibition showed a marked dependence on timing (Fig 3B). The interesting case (2) was observed in a constrained time window of delays between onset of somatic stimulation and onset of inhibition, which was about 1 ms (Fig 3B, light orange area). Given that bAPs are relatively short regenerative events that rapidly invade the dendritic tree, inhibition had to be available at the time of the spike. Additionally, early proximal inhibition could prevent the neuron from firing by suppressing action potential initiation in the axon initial segment, corresponding to case (3) (Fig 3B, black area).

Conforming with the local effects of shunting inhibition [21, 22], distal inhibition had no effect on bAPs in the oblique dendrites (S3B Fig).

#### Maintaining forward-directed signal flow from excitatory dendritic synapses

So far in the analysis, somatic action potentials depended on somatic current injection. The majority of excitatory inputs, however, arrive on the dendrites. For the hypothesized inhibitory regulation of plasticity not to interfere with neuronal processing, it needs to be ensured that shunting inhibition does not impair the forward flow of signals from the dendrites to the soma (while canceling the bAP). We hence also included conductance-based excitatory inputs to the dendrites.

In the simplest case, inhibition modulates dendritic signals of postsynaptic spiking that was triggered by an excitatory pathway arriving in a different dendritic compartment. This case proved largely identical to direct somatic stimulation, because inhibitory inputs are not on the electrotonic route from the site of excitatory stimulation to the soma. Functionally, postsynaptic activity triggered by synaptic input in one dendritic compartment A could potentially mediate plasticity in a different compartment B. Our results suggest that in this scenario, synaptic plasticity in dendritic compartment B can be readily modulated by inhibitory inputs, by controling the spread of dendritic signals in this compartment.

In a more complex setting, excitatory stimulation and inhibitory control impinge onto the same dendrite. In this case, it is less clear whether inhibition of backpropagating dendritic signals is possible without impairing the ability of forward-directed input integration. A quantitative analysis revealed that an EPSP could reach the soma despite an inhibitory conductance (located 90 μm from the soma) that was capable of suppressing the bAP within a time window of about half a millisecond (Fig 3C). The width of this window, however, depended on the location of inhibition and broadened with increasing distance of inhibition from the soma, swiftly exceeding 1 ms (S4 Fig). The farther inhibition was located from the soma, the more time remained between the passage of a forward-directed EPSP and the arrival of a bAP at the site of inhibition, resulting in an increase of the time window. Functionally, this scenario applies to the case where postsynaptic activity triggered by input onto a given dendritic compartment mediates plasticity in the same compartment. Our results suggest that an inhibitory modulation of this form of plasticity is possible, but requires a precise timing of inhibition.

### Inhibition of bAPs and calcium spikes during BAC firing

While many neurons exhibit bAPs, some neuron types (such as cortical layer 5 and CA1 pyramidal neurons) can additionally generate distal calcium spikes that trigger BAC firing. Because calcium spikes can also serve as plasticity signals [26], we analyzed the effects of inhibition on bAPs and calcium spikes during this firing mode. To trigger calcium spikes in the distal dendrite, we here combined somatic current injection in our model with coincident depolarization in the distal dendrite.

**Fig 4:**
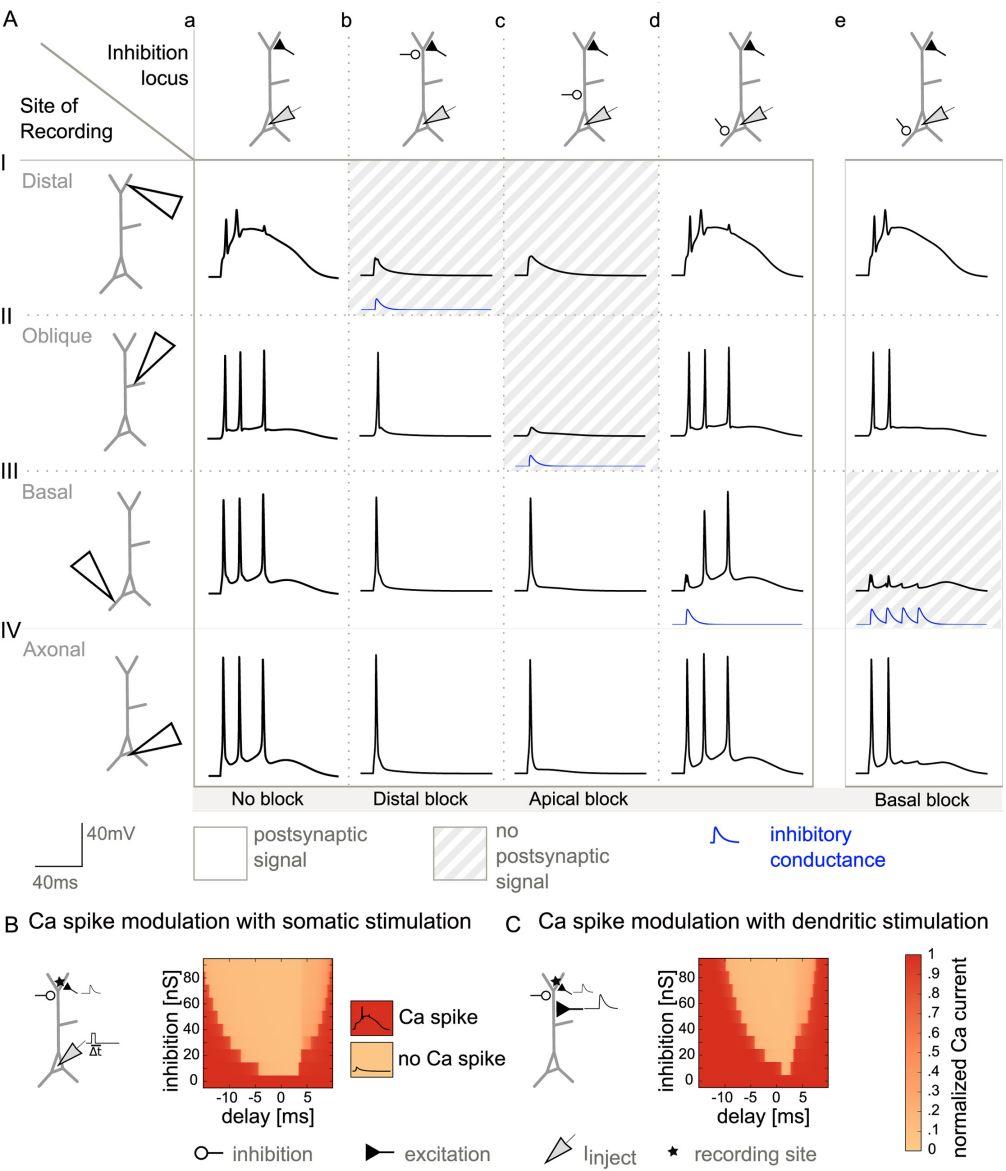
Compartment-specific inhibition of bAPs and calcium spikes during BAC firing. A: Effect of inhibition locus on dendritic coincidence signals, when somatic current injection was paired with dendritic excitation (with a delay Δt of 0 ms) to trigger a calcium spike (as in Fig 1D). As in Fig 3A, the recording site varies along the rows; the site of inhibition varies along the columns. Inhibitory conductance is indicated by the blue traces (inhibition onset was 1.5 ms after stimulation onset.). a: No inhibition. b: Distal inhibition (460 μm, 50 nS) suppressed the calcium spike (I) and thus distal plasticity, but left signaling in the remaining dendritic tree intact (II and III). c: Proximal inhibition (90 μm, 50 nS) affected signaling in the whole apical dendrite by eliminating the bAP (II), and thus the calcium spike (I), but did not affect the bAP in the basal dendrite (III). d: In the presence of a calcium-spike induced somatic burst, one inhibitory pulse was not sufficient to block the propagation of all bAPs into the basal dendrite (III). e: The train of bAPs in the basal dendrite, and thus basal plasticity, was suppressed by four inhibitory conductance changes at a frequency of 75 Hz on the proximal basal dendrite (100 μm, 70 nS) (III), while apical signaling was unchanged (I and II). B: Inhibition of calcium spikes in the distal apical dendrite. Calcium spikes were triggered by coincident bAPs and distal excitation with a temporal separation (Δt) of 0 ms (as in A). Color-coded is the calcium transient in the apical tuft, normalized to its uninhibited value. While the bAP could be modulated by proximal inhibition within a time window of 1 ms (Fig 3B), calcium spikes were rather insensitive to timing, and were abolished by weak distal inhibition. C: Inhibition of calcium spikes in the distal apical dendrite, when the neuron was driven by excitatory synapses distributed along the apical trunk to represent inputs from oblique dendrites (see Methods). The EPSPs were paired with distal excitation with a temporal separation (Δt) of 0 ms. As in B, the calcium spike could be modulated, less dependent on timing than the bAP.

In the control situation without inhibition (Fig 4A–a), a calcium spike was triggered in the distal dendrite and the resulting BAC-firing-induced bursts of APs could be observed in the other compartments. Calcium spikes (and the accompanying burst of APs) proved highly sensitive to inhibitory inputs in the distal part of the apical dendrite (Fig 4A–bI). In contrast, the bAP was relatively robust to distal inhibition and readily invaded both the apical oblique and basal dendrites despite the presence of distal inhibition (Fig 4A–bII/III). In the basal dendrite, inhibition was not sufficient to cancel all BAC firing related bAPs, because the single GABA_A_ conductance decayed too fast, and hence could not exert an influence on later bAPs (Fig 4A–dIII). bAPs in the basal dendrite could, however, be controlled by inhibition that outlasted the burst. Such inhibition could be mediated by inhibitory synapses of the same dynamics but an activation that is distributed in time (as it could be provided by bursting interneurons). Along these lines, four temporally spread out inhibitory inputs were able to cancel the bAPs in the basal dendrite (Fig 4A–eIII). The quantitative dependence of this effect on frequency and number of such repetitive inhibitory inputs is shown in S2 Fig. Altogether, we have seen that, in principle, inhibition can cancel coincidence signals in a manner that is selective between compartments.

#### Different time scales for bAP versus calcium spike modulation

Next, we explored the timing requirements for inhibition to cancel the coincidence signals in the BAC firing mode. Despite the distal depolarization, the width of the timing window for bAP modulation remained unchanged (S3 Fig). The time window where inhibition could cancel a calcium spike, however, was broader (>5 ms) than the corresponding window for bAP cancellation (∼1 ms). Compared to bAP modulation, smaller inhibitory conductances were sufficient to block calcium spikes in the distal tuft.

### Robustness to morphology

**Fig 5:**
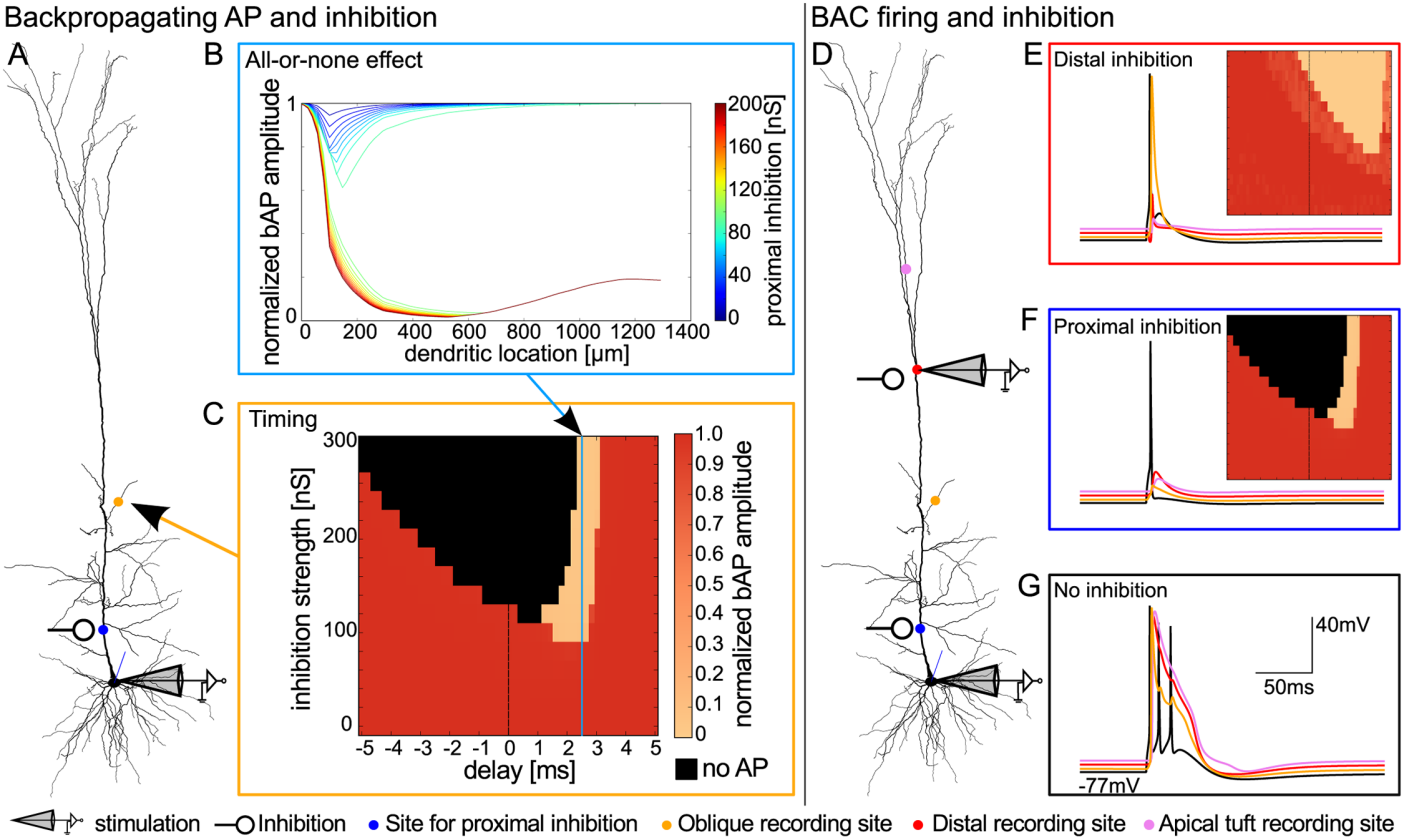
Validation of results in a model cell with anatomically reconstructed morphology. This model was previously fitted to account for BAC firing by Hay and colleagues [23]. Using their original parameters, we replicated the all-or-none modulation of bAPs by proximal inhibition (B), the compartmental modulation of bAPs versus calcium spikes in the morphologically complex dendrite (E-G), and the required time scales for proximal versus distal inhibition (C,E). Ionic conductances in the model by Hay et al. differed from those used in our simplified pyramidal cell model. We found the recovery of the bAP to be independent of the exact ion channel composition of the dendrite, as long as the interplay of sodium and counteracting currents allowed for an active propagation of the sodium spike. A: Morphology of the neuron. Somatic current injection as in [23] was used to elicit a bAP. Sites of proximal inhibition (blue) and oblique recording (yellow) are indicated. B: All-or-none modulation of the bAP along the apical dendrite, from the soma into the very distal apical tuft. C: Timing and strength of proximal inhibition (x- and y-axis, respectively) and its effect on the bAP amplitude in the oblique dendrite. Color-code as in Fig 3B/C. D: Somatic current injection paired with dendritic stimulation as in [23] was used to trigger BAC firing. Sites of proximal inhibition (blue), distal inhibition and recording (red), and apical tuft (pink) and oblique (yellow) recording are indicated and correspond to colored voltage traces in E to G. E: Distal inhibition inhibited BAC firing, but did not inhibit the bAP in the oblique dendrite. Inhibition of the calcium spike could be achieved for a range of timings (inset, same axes and color code as in B). F: Proximal inhibition inhibited the bAP in the oblique dendrite and BAC firing, if timed appropriately (inset). G: Without inhibition, pairing of bAP and distal input results in BAC firing [23].

To demonstrate the robustness of our results with respect to morphological and physiological detail, we replicated the main findings in an anatomically reconstructed L5 neuron model with different physiology [23] (Fig 5).

### Compartment-specific inhibition and plasticity

**Fig 6:**
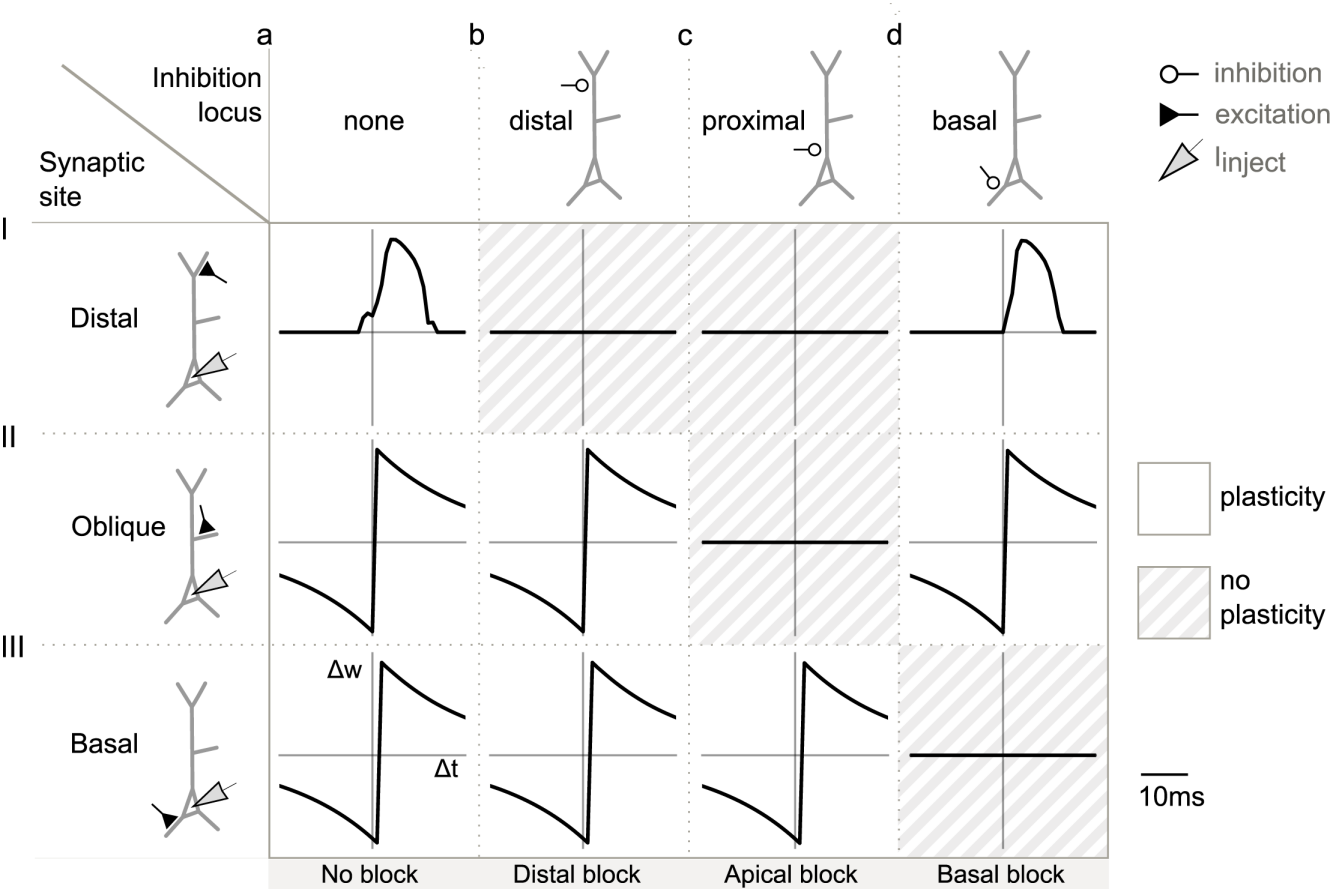
Switching STDP learning rules by inhibition of dendritic signals in a compartment-specific manner. Results are presented in the same format as in Fig 3A. For each synaptic location, a somatic step current was paired 100 times at 1 Hz with the synaptic activation at different Δt, to simulate a pairing protocol, and to measure the resulting plasticity rule. Inhibition was placed at different locations on the dendritic tree, inhibition onset was 1.5 ms after stimulation onset, rise and decay time constants were 0.5 ms and 5 ms, respectively, the maximum conductance amounted to 50 nS. Synaptic change was normalized to its maximum. A: No inhibition. B: Distal inhibition on the apical dendrite led to a flat STDP window in the distal synapse. C: Proximal inhibition on the apical dendrite caused zero synaptic change in both oblique and distal synapses. D: Proximal inhibition on the basal dendrite abolished STDP in the basal synapse.

To demonstrate that cancelation of the coincidence signals indeed results in the anticipated changes to synaptic plasticity, we next subjected the model cell to a classical plasticity paradigm. Contemporary biophysical and phenomenological models of STDP [13, 27–29] depend on postsynaptic variables such as depolarization or calcium concentration, which are in turn shaped by bAPs and calcium spikes. Because the latter can be abolished by properly timed and placed inhibition, we suggest that cancellation of these signals via inhibition will lead to a noticeable change in the predicted learning rule (or abolish plasticity altogether). To demonstrate this, we simulated a typical STDP pairing protocol (see, for example, [30, 31]). Somatic current injection was paired with excitatory synaptic activation on either basal, oblique or distal dendrites with a time delay Δt. Excitatory synaptic plasticity was Hebbian with a positive weight change for positive timings between the activation of the synapse and the arrival of a depolarizing postsynaptic potential and a negative weight change for negative timings (for details see Methods). We implemented the simple additive rule by [13], leading to the classical asymmetric STDP window (Fig 6). While the learning windows for oblique and basal synapses resembled the classical one (Fig 6II/III), we found the distal learning window to be more symmetric (Fig 6I). The latter results from the assumption that at distal synapses a calcium spike serves as the signal for plasticity. Because a calcium spike required the coincidence of bAP and EPSP, a calcium spike could only occur (see Methods) after presynaptic activation, such that the synaptic change was positive unless the relative timing of bAP and EPSP did not lead to a calcium spike (in which case it was zero). As expected, inhibitory cancellation of bAPs and calcium spikes in the dendrite resulted in flat learning windows, corresponding to zero synaptic change (compare Figs 3A, 4A and 6). Because of the all-or-none effect of inhibition on dendritic signals, we predict a switch-like effect on learning for any plasticity rule relying on the coincidence of pre- and postsynaptic spiking.

### How to satisfy the timing constraints for the inhibitory modulation of bAPs

**Fig 7:**
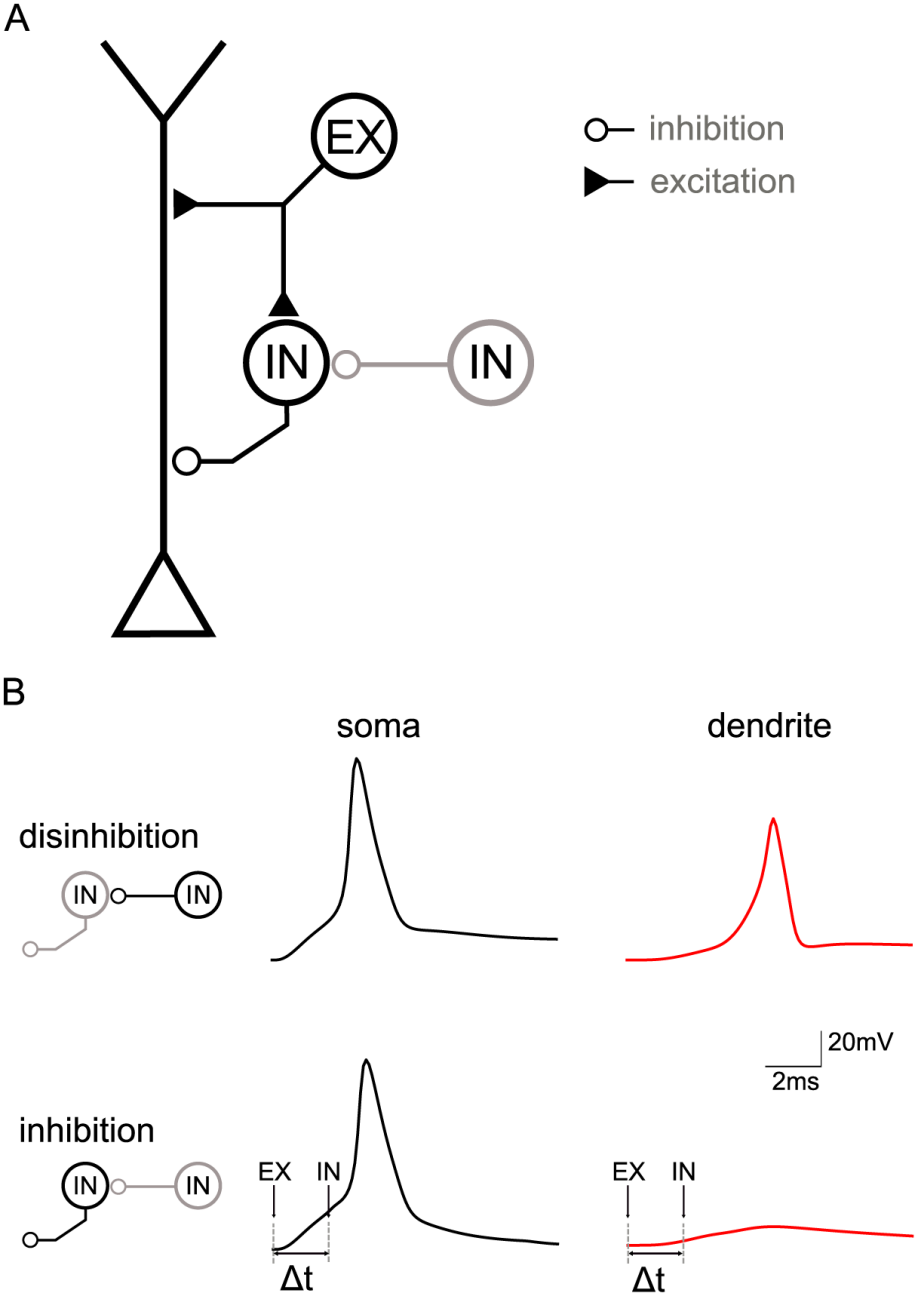
Circuit model of feedforward inhibition. A: Topology of the circuit with a multi-compartment pyramidal neuron model and a model of a fast-spiking inhibitory interneuron (IN, targeting pyramidal neuron) receiving excitatory input from a source (EX) at time t_0_ and potentially tonic disinhibition (IN, targeting IN). B: Effect of switching the feedforward interneuron on and off (via a second interneuron—marked gray in panel A) measured in the dendrite of the pyramidal neuron (370 μm from the soma), which in turn is driven by apical excitation. Note that the circuit is identical to that in panel A, although the lefthand schematics only zoom in on the interneurons. Upper paradigm: When the feedforward interneuron is switched off (in gray) because of activation of the second interneuron (in black), excitation triggers a spike in the pyramidal cell’s soma which propagates unhindered into the dendrite. Lower paradigm: When the feedforward interneuron is active (in black) due to excitation and because the second interneuron is switched off (in gray), excitation triggers a spike in the pyramidal cell’s soma that does not propagate far into the dendrite due to the inhibition provided by the feedforward interneuron (90 μm from soma, Δt = 2 ms).

Above, we have shown that the modulation of the bAP requires inhibition to fall into a small time window closely tied to the initiation of the somatic action potential in the pyramidal neuron. The question arises whether such timing is realistic and how it can be achieved. As a proof of principle, we here show that the common local circuit motif of feedforward inhibition [32] is a good candidate to provide the appropriate timing. In feedforward inhibition an excitatory signal is passed on to a pyramidal neuron along two parallel pathways: one direct excitatory pathway to the pyramidal cell and one indirect pathway, where the signal first excites an inhibitory neuron that sends its output on to the pyramidal cell (see Fig 7A, black parts of the schematic representation). From the perspective of the pyramidal neuron, excitation in this circuit arrives first to be followed by a delayed inhibition [33]. Embedding our pyramidal cell model into this circuit we found that inhibition arriving 2 ms after the EPSP was suited to control the bAP signal (Fig 7B, Δt = 2 ms). This delay matched experimentally measured inhibition delays well [33, 34] and was suited to control backpropagation without affecting forward-directed signal flow responsible for the initiation of postsynaptic somatic spikes.

Interestingly, interneuron dynamics play a role for the timing requirements. Dependent on interneuron type, spike latencies and firing frequencies can vary. The size of the temporal window where inhibition can cancel bAPs (without canceling EPSPs) tends to be smaller for proximal inhibitory synapses than more distal synapses, as outlined above. One may hence speculate that the dynamics of interneurons innervating proximal parts of the dendritic tree should be sufficiently fast. To highlight the role of interneuron dynamics, we probed the circuit with two interneurons differing in their spiking dynamics. We used the interneuron model from the previous paragraph and compared its performance in the circuit to that of a second interneuron with slower dynamics. The latter was implemented by artificially slowing down the opening and closing rates of the interneuron’s sodium channels. In this example, only the interneuron with a short spike latency met the tight timing requirements for bAP modulation at a more proximal synaptic location (S6A Fig). The timing provided by the slower interneuron did not suffice to cancel the bAP proximally. In contrast, both (faster and slower) interneurons were able to cancel the bAP (and accompanying calcium spike) when their synapses were located at more distal parts of the dendritic tree (S6B Fig).

## Discussion

In this study, we investigate how dendritic inhibition can serve to regulate Hebbian plasticity of excitatory synapses. Using multicompartmental biophysical neuron models, we show that shunting inhibition can gate the propagation of bAPs as well as dendritic calcium spikes in an all-or-none manner and thus exert direct control over the Hebbian coincidence signals. As a functional consequence, inhibition can provide a binary switch of synaptic plasticity. This switch can be specific for pathways in the local network as well as subsets of synapses within the same neuron. Importantly, forward-directed information flow via orthodromic EPSPs can be maintained when bAPs and calcium spikes are annihilated by shunting inhibition. The trivial scenario, where inhibition cancels not only Hebbian coincidence signals but also the excitatory drive of the neuron, can hence be avoided. Quantitative analyses of the physiologically constrained models reveal that this mechanism imposes strict timing constraints: while for the regulation of bAPs inhibition has to fall into a specific time window of a width of 1 ms, regulation of calcium spikes requires less precise timing. The time scales for proximal and distal inhibitory modulation of plasticity differ by several milliseconds. Finally, we suggest and provide a proof of principle that the precise timing required for bAP modulation can be achieved by a local circuit including the common network motif of feedforward inhibition.

### A functional role for inhibition in the context of plasticity

The central hypothesis that inhibition can control synaptic plasticity has been discussed in the experimental and theoretical literature [5–7, 35–37]. Its feasibility and functional relevance relate to three observations. First, different compartments within a neuron often receive excitatory input from distinct synaptic pathways [15], such that a compartment-specific regulation of plasticity could be functionally advantageous. Second, different compartments are targeted by different inhibitory interneuron classes [25, 38], so that Hebbian coincidence signals could be regulated locally. Third, dendrites support different plasticity-related coincidence signals, namely bAPs [39, 40] and calcium spikes [11, 18, 26], which are sensitive to inhibitory control [4, 11, 41–43]. Besides a switch in plasticity, inhibition has been shown to contribute to the shape of temporal requirements for plasticity in different parts of the dendrite [44–46]. Moreover, the induction and coincidence requirements of plasticity have been described to change with development, potentially through an increase in inhibition [47–49].

Strong experimental evidence in support of the ability of inhibition to modulate plasticity via gating of bAPs was recently provided by Müllner et al. (2015) [8]. They showed that one important step in this mechanism—the suppression of somatically elicited bAPs by dendritic inhibitory synapses with high temporal precision—is indeed possible. For a robust switch of plasticity, however, several points remain to be shown: (1) gating of coincidence signals can be exerted in a controlled and systematic manner, (2) forward information flow via EPSPs can be maintained, and (3) plasticity itself is altered. Our analysis on the basis of mathematical models with physiologically constrained properties demonstrates that all three points can be fulfilled. In particular, we find that shunting inhibition is sufficient to cancel bAPs and calcium spikes while preserving the ability of EPSPs to elicit somatic APs. Timing of inhibition needs to be precise, but is not unrealistic (∼1 ms for bAPs and >5 ms for calcium spikes).

### Model-derived predictions

Our study makes several testable predictions. In particular, the all-or-none nature of bAP modulation enabling the binary switch as well as the compartment specificity could be tested in experiments. To investigate the latter, classical paired recording paradigms for synaptic plasticity could be extended by optogenetic stimulation of different interneuron classes. Particularly promising candidates are the above mentioned SOM or PV interneurons that target perisomatic and distal dendritic regions of pyramidal cells, respectively [25, 38].

Moreover, perisomatic versus distal modulation of dendritic coincidence signals poses different timing requirements on inhibition. The proximity of inhibition to the soma constrains the modulation window, because the times of passage of the forward-directed EPSP and the backward-directed bAP (at the location of the inhibitory synapse) are very close. Therefore, inhibitory synapses ought to have a certain distance to the soma to be well suited to control the bAP and plasticity without canceling somatic spiking. Our results suggest that at a distance of ∼100 μm, which is relatively proximal for apical dendrites, the timing window becomes wide enough to enable inhibitory control (S4 Fig). We predict that the regulation of bAPs caused by more proximal excitation in the pyramidal neuron may be better achieved by interneurons with short latencies, such as fast-spiking interneurons. We found that distal inhibition of the calcium spike did not require very precise timing and tolerated longer delays to the onset of shunting inhibition. In the context of the here discussed mechanism of plasticity regulation, it would be functionally useful if proximal and distal inhibition were accomplished by interneurons of different dynamics. This proposition is in line with the fact that the many interneuron types connected to pyramidal cells differ in their spiking dynamics as well as their dendritic target location in pyramidal cells. Regulation of calcium spikes in the distal dendrite, on the other hand, need not be provided by local circuits, but could be mediated by longer-range connections including multiple synaptic transmissions. One may speculate that this time scale is beneficial when incorporating top-down information that tends to arrive in superficial layers [50].

From the perspective of the pyramidal neuron, we find that, a short rise time of synaptic inhibition (like that typical for GABA_A_-mediated inhibition (∼0.5 ms)) is crucial for the mechanism to operate effectively. In contrast, the temporal extent of inhibition (determined by the temporal extent of inhibitory input as well as the decay time constant of inhibition) is less important. These variables are likely to be more relevant in setting a lower frequency limit to excitatory signals because they could interfere with a following EPSP if inhibition lasted for too long. For GABA_A_-typical decay time constants on the order of 5 ms (as used here), such limits to the frequency of EPSPs are sufficiently large (>100 Hz). We note that GABA_A_-mediated inhibitory postsynaptic potentials (IPSPs) have been described to differ between proximal and distal sites [20]. The difference is mainly in half-width and decay time constant, less in rise time. We hence used the same time constants for proximal and distal GABA_A_-mediated currents.

Our results suggest that, in contrast to the other compartments, in basal dendrites several volleys of interneuron input may be needed to suppress all bAPs. In this scenario, a burst of bAPs invades the dendrite in the BAC firing mode. For this compartment, an innervation by bursting interneurons may hence be advantageous and several bursting interneuron types, including double bouquet cells [51] and bistratified cells [52–54], have been described.

Our prediction on the timing dependence of the modulation of bAPs quantitatively agrees with the experimental study by [8]. They found that calcium transients, evoked by a train of bAPs, are maximally inhibited with a spike timing (between interneuron and pyramidal cell) on the order of < 5 ms. This time scale is compatible with our predictions for the required timing of bAP modulation (see Fig 3B/C). Also space constants of inhibition, observed to be on the order of 23–28 μm by Müllner and colleagues, are comparable to our results (see Fig 2).

### Implications of feedforward inhibition

As our study showed, inhibition has to fall into a narrow time window to gate APs without simultaneously canceling EPSPs that are meant to drive the postsynaptic cell, potentially casting some doubt on the robustness of the mechanism of an inhibition-mediated plasticity switch. Feedforward inhibition, however, seems a good candidate to guarantee the appropriate timing, in particular, as in such a circuit inhibition follows excitation within a relatively fixed time interval. Feedforward inhibition is a common circuit motif [55, 56] that has, for example, been implicated in keeping a balance of excitation and inhibition and to open time windows for precise firing events [33, 34, 57]. While we do not suggest that it is the only mechanism that can provide suitable timing for the plasticity switch discussed here, it can satisfy both temporal requirements for the cancellation of bAPs: the delay between forward-directed excitation and the shunting inhibition, as well as the temporal precision on the order of a millisecond. In the circuit model (Fig 7A, black part of the schematic), a delay on the order of 2–3 ms between onset of the excitatory EPSP and the onset of the inhibitory IPSP in the pyramidal neuron was required to cancel the bAP triggered by the EPSP. This delay has to be accounted for by the processing in the inhibitory neuron itself and comprises the time the EPSP *in the interneuron* needed to reach this neuron’s soma, spike generation in this cell, propagation of the action potential along the axon, and the inhibitory synaptic transmission between interneuron and pyramidal cell. The timescale of 2–3 ms is plausible for these processes and agrees with the range reported in experiments [33, 34]. Characteristics of the interneuron allow for some flexibility of this delay [58]. For example, a fast spike generation (as in fast-spiking interneurons, due to lower firing threshold and/or stronger excitation on interneurons [59, 60]) plays an important role in keeping the delay short. Additionally, dendritic propagation is slower than axonal propagation, so that the relative length of these cables influences the required delay (which may potentially be correlated to the somatic location of the interneuron, assuming a regular, bipolar morphology where the soma lies in between dendrites and axon). For example, a more proximal excitation in the pyramidal neuron requires a shorter delay of inhibition. The integration time in the pyramidal neuron depends on the location, and distribution of synaptic excitatory inputs, next to being negatively proportional to the number, strength and rise time of the synaptic conductances.

In a feedforward inhibitory circuit, inhibition, by default, comes along with excitation. This means that an additional source is required to switch off the inhibitory influence (see Fig 7A, gray part of the schematic). *Per se*, when the interneuron is *active*, plasticity of excitatory pyramidal synapses is switched off. In turn, *silencing* the interneuron up-regulates pyramidal cell plasticity. This design indicates a disinhibitory regulation of Hebbian plasticity, which is in line with recent findings for behavioral learning [61, 62]. Interestingly, advances in unraveling the connectivity profile of different interneuron classes suggest that the cortical mircocircuitry seems to be well suited for a disinhibitory and compartment-specific regulation [24, 36, 63–65]. In particular, vaso-intestinal peptide (VIP) expressing interneurons and other supragranular interneuron classes have been proposed to modulate the activity of somatostatin (SOM)- and parvalbumin (PV)- positive cells—inhibitory interneurons with distinct postsynaptic targets within the pyramidal dendrite—in a way that is consistent with a rapid redistribution of inhibition between perisomatic and distal apical dendrites of pyramidal cells [24, 66].

### Other forms of synaptic plasticity

We note that distal disinhibition in pyramidal cells is special because it can significantly and reversibly increase the occurrence of calcium spikes and somatic bursts [67]. Calcium-induced bursting has been proposed as a mechanism for the association of inputs arriving through different pathways [11]. Because synaptic plasticity is often more efficiently induced by pairing presynaptic inputs with postsynaptic bursts, it is tempting to speculate that calcium spikes can induce a form of global synaptic plasticity within a neuron. Consequently, plasticity in the entire dendritic tree could be regulated at a single spot through local disinhibition. Note that such a form of plasticity regulation does not exclude, but complements our main hypothesis, because proximal inhibition could contribute to impair the arrival of bursts of backpropagating APs at basal and oblique synapses (see Results on basal signaling). However, our prediction that basal plasticity regulation requires more complex inhibitory innervation in the presence of a calcium spike, possibly indicates that calcium-dependent burst firing is in place to overcome the effects of inhibition by increasing the likelihood of backpropagating action potentials passing the barrage.

STDP is an important and commonly observed mechanism underlying many forms of learning [68]. Regulation of STDP (a potential mechanism is considered in this study) is hence highly relevant. However, there are other, non-Hebbian forms of synaptic plasticity which are independent of postsynaptic spiking. For example, plasticity can be directly triggered by spikes of dendritic origin that arise locally from cooperative or strong synaptic activation [26]. The proposed pathway-specific switch does not apply to these types of plasticity. Still, theoretical and experimental studies have shown that dendritic spikes can be affected by inhibition on a local (spine- or branch-specific) or global level [69–71], possibly providing a mechanism to control timing-independent plasticity. Especially NMDA spikes cannot be excluded as a target for inhibitory modulation. Because they present a more local phenomenon that carries limited information about somatic spiking, an investigation thereof is beyond the scope of the present study. One kind of synaptic plasticity which is unlikely to be regulated by inhibition of dendritic events at all is presynaptically induced and expressed LTD [72].

### Conclusion

In this study, we provide computational evidence that the known physiological characteristics of pyramidal cells are sufficient to exert a binary control of plasticity while preserving excitatory, forward-directed information flow. Despite the fact that the identified timing requirements for this mechanism may at first seem tight, the proposed local circuit motif of feedforward inhibition seems well suited to provide inhibition at appropriate times. Our modeling work substantiates the point of view that inhibition is likely to play a crucial role for Hebbian plasticity of excitatory synapses in a manner that can be specific to individual pathways of the local network [5]. Recent advances of optogenetic methods allow to shed further light on the computational relevance of interneuron diversity [50, 63, 64, 73] and could—by targeting specific interneuron types—help to reveal whether (and if so which) neurons can regulate plasticity in local circuits.

## Methods

### Simplified morphology model

#### Morphology and passive parameters

Simulations were performed in NEURON [74], with an integration time step of 0.1 ms and a temperature of 30°C. The neuron consisted of an axon initial segment (2 μm in diameter, 3 μm in length, nseg = 1), soma (diameter and height 18.5 μm, nseg = 1), an apical dendrite, and a basal dendrite (Fig 1A). The apical dendrite had a main shaft (diameter 2 μm), first- and second-order branches (2/3 of the diameter of their respective mother branch). In addition to the distal branches, an oblique dendrite was joined to the apical trunk 100 μm from the soma, dividing the trunk into two compartments, a 100 μm long compartment (nseg = 19) and a 400 μm long compartment (nseg = 73). Its total length hence amounted to 500 μm, while all other branches had a length of 300 μm (nseg = 91). Basal dendrite parameters were: main shaft diameter 1 μm, daughter branches 2/3 of their mother branches, length 150 μm for all basal dendrites (nseg = 91). Second- order branches were added to prevent boundary effects, but not specifically studied; they are not shown in the schematic figures. The passive parameters of the neuron were: membrane capacitance C_m_ = 0.75 μF/cm^2^, axial resistivity R_a_ = 150 Ωcm, and membrane resistance R_m_ = 40,000 Ωcm^2^; leak reversal potential E_L_ = -70 mV. The simulation code is available in the ModelDB database under the accession number 187603 (https://senselab.med.yale.edu/ModelDB/).

#### Active conductances

Both the neuron and its dendrites hosted active conductances to allow somatic and dendritic action potentials. The voltage-dependent conductances were limited to those needed for the occurrence of backpropagating action potentials and calcium spikes as experimentally observed. In a first step, inactivating sodium and delayed-rectifying potassium conductances with channel kinetics from [75] (ModelDB accession 2796) were adjusted in density such that bAPs propagated within the dendritic tree with a stable amplitude. The spatial distribution of these conductances was uniform [76]. To account for the attenuation of the bAP [77], A-type potassium channels (ModelDB accession 2796, kaprox.mod, [75]) were added in a second step, with linearly increasing density from soma to distal dendrites. The three conductances and the spatial distribution of the A-type potassium channels were fitted such that the model output matched experimentally observed bAP attenuation in CA1 pyramidal cells [10]. Dendritic sodium and delayed-rectifier potassium channels were distributed uniformly with a maximum conductance of 0.009 S/cm^2^ and 0.01 S/cm^2^, respectively. The voltage dependence of dendritic sodium channel activation was shifted by +5 mV to allow apical excitatory postsynaptic potentials (EPSPs) to cause somatic spikes without eliciting dendritic spikes. The reversal potentials were: E_Na_ = +60 mV and E_K_ = -80 mV. The A-type potassium conductance increased five-fold up to 500 μm from the soma with an initial value of 0.029 S/cm^2^.

To account for calcium spikes, a calcium spike initiation zone with increased calcium channel densities in the apical tuft between 500 and 750 μm from the soma was incorporated. While high-voltage activated calcium channels (Ca,H), a calcium-activated potassium current (K,Ca) and a calcium decay mechanism were present in all dendritic compartments, we added low-threshold T-type calcium channels (Ca,L) only to the active zone. All channel kinetics were taken from [78] (ModelDB accession: 83344), who adapted the calcium currents from the pyramidal cell model of [79]. The dendritic channel distributions outside the spike initiation zone were (in S/cm^2^): g_Ca,H_ = 0.00015, g_K,Ca_ = 0.00025 and inside: g_Ca,H_ was increased 3-fold, g_Ca,L_ = 0.005. In the soma, g_Ca,H_ and g_K,Ca_ were increased 2-fold compared to the dendrite. The reversal potential for calcium was E_Ca_ = +140 mV.

#### Axonal spike initiation zone

The axon initial segment consisted of only a single compartment with increased sodium channel density compared to dendritic compartments (0.6 S/cm^2^ in total), matching the order of magnitude of the experimentally measured value (2,500 pS/μm^2^ = 0.25 S/cm^2^) based on modern sodium imaging techniques [80]). Voltage dependencies were shifted in 50% of the axonal sodium channels by -10 mV [81–84].

#### Stimulation protocol and synaptic inputs

If not otherwise indicated, the simplified model was stimulated by somatic step current injection of 0.3 nA for 2 ms. All synapses were modeled as time-dependent conductance changes. The dendritic synapse in Fig 1 (530 μm) had a double exponential time course (a NEURON Exp2Syn synapse with rise and decay time constants of 0.5 and 2 ms, respectively). A maximum conductance of 8 nS was required to trigger a calcium spike in the presence of a bAP. The stronger synapse in Fig 1E had a maximum conductance of 14 nS. In Figs 3C and 4C, the neuron was driven by eight excitatory synapses (Exp2Syn) of the same type distributed equally between 140 and 420 μm, i.e. between the oblique dendrite and the main branching site, representing inputs arriving from putative oblique dendrites. The total maximum conductance of these synapses amounted to 20 nS. Plastic dendritic excitatory synapses in either the basal dendrite (middle of main shaft: 75 μm), the oblique dendrite (middle: 250 μm), or the apical tuft (530 μm) had an exponential time course with reversal potential 0 mV and time constant 3 ms. As in Fig 1, the apical tuft synapse had a maximum conductance of 8 nS as this was required to trigger a calcium spike. The other plastic synapses were initiated with small maximum conductances of 0.001 nS.

Inhibition was modeled to resemble GABA_A_-mediated, and not GABA_B_-mediated inhibition, because the former is more abundant, and its time constants are better suited for the task. The large time constants of GABA_B_ mediated inhibition are not well-suited, because they lead to (1) slow inhibition, which cannot fulfill the tight timing constraints for bAP inhibition, and (2) long-lasting inhibition, which, next to being a waste of energy, can interfere with subsequent signaling. GABAergic inhibition had a double-exponential time course (NEURON Exp2Syn synapse; rise and decay time constants 0.5 and 5 ms, respectively). The reversal potential of the inhibitory synapses was close to the resting potential, i.e. -73 mV. At rest, inhibition hence did not hyperpolarize the membrane potential, but acted as a local “shunt”, i.e. increased the membrane conductance. The maximum conductance, referred to as inhibition strength, and the onset of inhibition was varied as indicated.

For control, inhibitory synapses were distributed in space and time according to a Gaussian distribution with varying standard deviation sigma. The spatial distribution was centered around 90 μm and truncated such that synapses were restricted to the apical trunk. The temporal distribution of inhibition onsets was centered around 2 ms after the onset of somatic stimulation.

### Spike detection

To study the impact of inhibition on dendritic spikes, we recorded the membrane voltage in the basal dendrite (75 μm), the oblique dendrite (370 μm) and the apical tuft (650 μm). Additionally, we monitored the calcium current in the apical tuft at the same spot. Spike amplitudes were measured as the maximum voltage deviation from rest (a membrane voltage reaching 10 mV would be a spike of amplitude 10 mV-(-73 mV) = 83 mV). bAPs were normalized to the non-inhibited amplitude of the first bAP. Calcium spikes were quantified by the integral of the calcium current (to clearly disambiguate between calcium and sodium components of the voltage trace at the same spot), normalized to the non-inhibited calcium current. A somatic spike was detected when its amplitude reached a threshold of 80 mV.

### Synaptic plasticity and pairing protocol

Synaptic plasticity was implemented with an additive spike-timing dependent plasticity rule as in [13]:

$$\begin{array}{c} \Delta w=\{\begin{array}{cc} -A_{-}\exp\left(\frac{\Delta t}{\tau_{-}}\right) & \text{if}\;\Delta t\leq 0\\ A_{+}\exp\left(-\frac{\Delta t}{\tau_{+}}\right) & \text{if}\;\Delta t>0 \end{array} \end{array}$$

where Δ*t* is the difference between pre- and postsynaptic spike time: t_post_-t_pre_. A postsynaptic spike was counted when the membrane voltage at the synapse reached a threshold of -20 mV. For apical tuft plasticity, a postsynaptic spike was counted when the calcium concentration reached a threshold of 0.5 mM. We used a different model for distal plasticity, assuming that the calcium spike governs calcium dynamics and thus plasticity in the distal dendrite (for an illustration of our argument, see S5 Fig). Parameters were: potentiation factor *A*_+_ = 0.001, depression factor *A*_−_ = 0.00106, potentiation time constant *τ*_+_ and depression time constant *τ*_−_ were both 20 ms. Hard bounds set to 0 and 0.0001 μS were imposed on synaptic weights.

Somatic current injection was paired with dendritic synapse activation 100 times with a frequency of 1 Hz with varying Δ*t*.

### Circuit model

The feedforward inhibitory circuit contained the introduced multi-compartment pyramidal neuron model and a model of a fast-spiking interneuron (IN). The latter, a single-compartment model with Hodgkin-Huxley-type sodium and potassium conductances, was taken from [85] (ModelDB accession: 3817). The interneuron with slower spike initiation was adapted from the fast-spiking interneurons by changing the sodium dynamics. The opening rate α was changed by a factor of 0.1, the closing rate β by a factor of 0.2. Both, the interneuron and the pyramidal neuron received EPSPs (NEURON Exp2Syn with rise and decay time constants of 0.5 ms and 2 ms, respectively, and a reversal potential of 0 mV) from an excitatory source (EX) at time t_0_. The excitatory synapse onto the interneuron had a maximum conductance of 300 nS. To mimic inputs from oblique dendrites, eight synapses were distributed onto the apical trunk of the pyramidal neuron between 140 and 420 μm [86] with a total maximum conductance of 20 nS. The inhibitory synapse from the fast-spiking interneuron had the same properties as all shunting synapses modeled in this study (τ_rise_ = 0.5 ms; τ_decay_ = 5 ms; reversal potential -73 mV).

### Detailed L5 model

From [23], we took the neocortical L5 pyramidal cell model constrained by both BAC firing and perisomatic step current firing with an anatomically reconstructed morphology (ModelDB accession: 139653). We did not change any parameters. To investigate the all-or-none effect, the model was stimulated with somatic current injection as in [23]. We added inhibitory synapses (NEURON Exp2Syn synapses; rise and decay time constants 0.5 and 5 ms, respectively) to the model and placed them ∼ 100 μm (proximal) and ∼617 μm (distal) from the soma. The onset of inhibition was 2.5 ms after the onset of the somatic pulse if not varied. Voltage traces were measured in the soma, the oblique branch and an apical tuft branch. In Fig 5E and 5F, inhibition had maximum conductances of 100 nS (proximal) and 200 nS (distal).

## Supporting Information

S1 Fig Spatial spread of inhibition. Inhibitory synapses were normally distributed around a mean of 90 μm from the soma with varying standard deviation *σ* up to 50 μm. The effect on the normalized bAP amplitude is shown as a function of the number of inhibitory synapses, each with a maximum conductance of 1 nS.
(EPS)

S2 Fig Basal modulation in the presence of a calcium spike. Modulation of bAPs propagating into the basal dendrite when a calcium spike was triggered by concurrent somatic current injection and dendritic excitation (with a delay Δt of 0 ms, as in Fig 4A). Shown (color-code) is the maximum bAP amplitude measured in the basal dendrite 75 μm from the soma as a function of basal inhibition. The number of spikes in the inhibitory spike train vary along the horizontal axis, the frequency with which the inhibitory neuron fired varies along the vertical axis. Higher frequencies than 75 Hz (chosen in Fig 4A) were also sufficient to inhibit the train of bAPs with the same amount of synaptic discharges. However, generally holds that with higher frequency more synaptic discharges are needed to cover the duration of the burst.
(EPS)

S3 Fig Timing requirements—additional plots. Results are in the same format as in Fig 3B/C (color-code and axes are the same). A: Proximal inhibition on the apical dendrite and its effect on bAPs (top) and calcium spikes (bottom), when the neuron was stimulated by somatic current injection and dendritic excitation (with a delay Δt of 0 ms). When proximal inhibition abolished the bAP, it also affected the generation of a calcium spike. B: Distal inhibition on the apical dendrite did not affect bAPs in the oblique dendrite.
(EPS)

S4 Fig Width of the timing window as a function of inhibitory location. Timing requirements for bAP modulation as a function of the dendritic location of inhibition. The simulation paradigm is identical to that in Fig 3C (dendritic stimulation). Color-coded is the amplitude of the bAP measured in the oblique dendrite as a function of the strength and timing of proximal inhibition. Unless somatic spiking was inhibited (black), either a full-blown bAP (red) or no bAP (light orange) could be observed. Location of inhibition was varied; distance to soma increases from left to right, as marked. All other parameters as in Fig 3C. The width of the modulation window (light orange area) increased with distance of the inhibitory synapse from the soma and exceeded 1 ms for locations more distal than or equal to 150 μm.
(EPS)

S5 Fig The distribution of distal calcium concentration is bimodal. BAC-firing and the corresponding distal calcium influx is triggered if pre- and postsynaptic spike times are coincident. Therefore, the amount of calcium, quantified by the time integral of the distal calcium concentration (A) or its maximum amplitude (B), is dependent on the time difference Δt (top panel in A and B, Δt varies between -20 and 20 ms). Because the calcium spike is a nonlinear event, the amount of calcium is bimodally distributed (bottom panel in A and B), such that BAC firing governs calcium dynamics in the distal dendrite. Therefore, a threshold for plasticity based on the calcium concentration could be easily set by locating it in between the peaks of the distribution (dashed line in B bottom). In the presence of (proximal or distal) inhibition, the amount of calcium was low, regardless of Δt, because BAC firing was prevented (top middle and right in A and B, bottom panel in A and B). For both proximal and distal inhibition, the amount of calcium was below the chosen plasticity threshold (bottom in B).
(EPS)

S6 Fig Example of the effect of inhibition onto the bAP amplitude for two different versions of an interneuron (with faster and slower dynamics, respectively). Both interneuron types were implemented in the feedforward circuit (see Fig 7). The normalized maximum amplitude of the bAP in the distal dendrite was monitored as a function of the inhibition strength. A: Two interneuron types with different spike latencies were placed on the proximal dendrite (at 90 μm from the soma). The fast interneuron could eliminate the bAP when sufficiently strong, while the slower interneuron fired too late to have an impact onto the bAP. B: The same two interneuron types were placed on the distal dendrite (at 460 μm from the soma). Both interneurons were properly timed to inhibit the distal calcium spike. For further details see main text.
(EPS)

S1 TableParameters of the simplified morphology model.s-soma, d-dendrite, a-apical calcium spike initiation zone, ax-axon. For ${\bar{g}}_{\text{K,A}}$, only the somatic value is given. But A-type potassium channels were present in all dendritic compartments, their density linearly increased 5-fold up to 500 μm from the soma. ${\bar{g}}_{\text{Na}}$ and ${\bar{g}}_{\text{K}}$ were uniformly distributed, only in the axon, ${\bar{g}}_{\text{Na}}$ had a different value.(PDF)
