# Supplementary material for: Inhibition as a Binary Switch for Excitatory Plasticity in Pyramidal Neurons
Source: PLoS Comput Biol. 2016 Mar 22;12(3):e1004768. doi: 10.1371/journal.pcbi.1004768 (PMC4803338; doi:10.1371/journal.pcbi.1004768)
Supplement: S1 Table — s-soma, d-dendrite, a-apical calcium spike initiation zone, ax-axon. For g¯K,A, only the somatic value is given. But A-type potassium channels were present in all dendritic compartments, their density linearly increased 5-fold up to 500 μm from the soma. g¯Na and g¯K were uniformly distributed, only in the axon, g¯Na had a different value. (PDF) [file pcbi.1004768.s007.pdf]

Table 1. Parameters of the simplified morphology model.

**Parameter**	**Value**	**Parameter**	**Value**
C_m	$0.75\,\mu F/cm^2$	$s.\bar{g}_{K,A}$	$0.029\,S/cm^2$
R_a	$150\,\Omega cm$	$s.\bar{g}_{Ca,H}$	$0.00030\,S/cm^2$
R_m	$40{,}000\,\Omega cm^2$	$s.\bar{g}_{K,Ca}$	$0.0005\,S/cm^2$
E_{leak}	-70 mV	$d.\bar{g}_{Ca,H}$	$0.00015\,S/cm^2$
E_{Na}	+60 mV	$d.\bar{g}_{K,Ca}$	$0.00025\,S/cm^2$
E_K	-80 mV	$a.\bar{g}_{Ca,H}$	$0.00045\,S/cm^2$
E_{Ca}	+140 mV	$a.\bar{g}_{K,Ca}$	$0.00025\,S/cm^2$
$\bar{g}_{Na}$	$0.009\,S/cm^2$	$a.\bar{g}_{Ca,L}$	$0.005\,S/cm^2$
$\bar{g}_K$	$0.01\,S/cm^2$	$ax.\bar{g}_{Na}$	$0.6\,S/cm^2$

s-soma, d-dendrite, a-apical calcium spike initiation zone, ax-axon. For $\bar{g}_{K,A}$, only the somatic value is given. But A-type potassium channels were present in all dendritic compartments, their density linearly increased 5-fold up to 500 μm from the soma. $\bar{g}_{Na}$ and $\bar{g}_K$ were uniformly distributed, only in the axon, $\bar{g}_{Na}$ had a different value.
